# A Systematic Review of Historical Temperature Data Use in Citrus Quality Assessment for Export Supply Chains

**DOI:** 10.3390/foods15071122

**Published:** 2026-03-24

**Authors:** Makhosazana Ngwenya, Leila Goedhals-Gerber, Louis Louw

**Affiliations:** Department of Industrial Engineering, Stellenbosch University, Stellenbosch 7602, South Africa; 18972586@sun.ac.za (M.N.); louisl@sun.ac.za (L.L.)

**Keywords:** cold chain management, postharvest losses, shipment records, temperature monitoring, digital twin technology, supply chain monitoring, shelf-life prediction

## Abstract

Global citrus exports rely heavily on temperature-controlled logistics to safeguard fruit quality and minimise postharvest losses. Temperature management remains a critical factor governing citrus quality throughout export logistics. Yet the extent to which historical shipment temperature data can meaningfully predict fruit condition at arrival has never been systematically assessed. This study presents a comprehensive review of how historical temperature records have been used to assess citrus quality within export supply chains, highlighting the lack of longitudinal temperature–quality correlations in existing research. Using PRISMA 2020 guidelines and Kitchenham’s three-phase review framework, 35 relevant peer-reviewed articles published between 2013 and 2025 were analysed. Bibliometric mapping identified dominant research concentrations in experimental cold chain studies and simulation-based approaches, with emerging themes around digital twins and virtual cold chain technologies. The review shows that current research predominantly employs controlled experimental designs and computational simulations to quantify temperature-driven deterioration, including chilling injury, decay rate, and weight loss. Although real-time temperature monitoring in commercial shipments is emerging, temperature deviations are rarely assessed alongside direct quality metrics. Although several studies have examined shipment temperatures alongside arrival-quality outcomes, these analyses are generally limited in duration, scope, or sensor resolution. Consequently, rigorous, multi-year, longitudinal datasets that pair detailed shipment temperature histories with standardised fruit-quality assessments remain largely unavailable, constraining the empirical validation of temperature–quality relationships in real export conditions. This gap significantly limits predictive capability in real-world export contexts. The review highlights the urgent need for a coordinated, long-term data infrastructure that integrates temperature and quality measurements across global citrus supply chains. Establishing such datasets, particularly in major exporting regions such as South Africa, would enable more robust modelling of temperature impacts, support the optimisation of cold chain practices, and contribute to international food loss-reduction goals.

## 1. Introduction

### 1.1. Citrus Global Trade

The global citrus fruit sector continues to exhibit robust production and trade performance, underpinned by regional comparative advantages and shaped by persistent challenges, including plant diseases, climate variability, and evolving market dynamics. In 2024, global citrus exports were valued at USD 16.3 billion, totalling 17.1 million tons and averaging USD 957 per ton according to International Trade Centre [1]. However, the 2024 export quantities declined by approximately 1%, suggesting a contraction in traded volumes despite stable monetary values. This discrepancy could reflect pricing shifts or changes in market demand, as the global trade balance for citrus products has remained in a persistent deficit of USD 1.48 billion, driven by a higher import dependency relative to export performance [1]. Further complicating the supply landscape, historical production challenges have been well-documented. For example, citrus greening (Huanglongbing) has significantly impacted orange production in the United States, especially in Florida, reducing output to approximately 2.1 million tons [2]. Similarly, adverse weather conditions in Türkiye impaired production growth. Consequently, production was forecasted to drop by nearly one-third to 1.6 million tons due to unfavourable weather during the bloom, resulting in lower yields, as noted in recent agricultural analyses [2].

**Figure 1:**
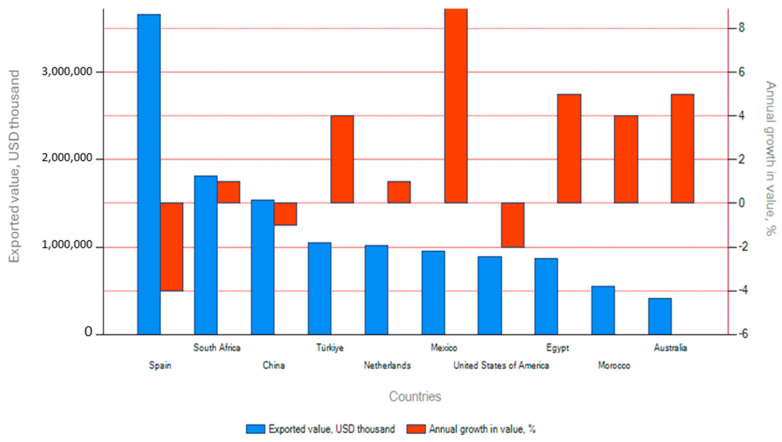
The export value (in thousand USD) and annual growth rate (in %) of citrus fruit, fresh or dried (product code 0805), for the top exporting countries in 2024. Sources: ITC calculations based on UN COMTRADE and ITC statistics.

Figure 1 illustrates the top citrus exporters by value, with Spain maintaining its lead in 2024 at USD 3.66 billion, despite experiencing negative growth in both short-term (1% between 2023 and 2024) and long-term (1% from 2020 to 2024) trade value. South Africa ranked second globally in citrus export value (USD 1.81 billion) and third in volume (2.54 million tons) in 2024. Although it recorded a 3% decline in value between 2023 and 2024, it achieved modest cumulative growth between 2020 and 2024, with a 1% increase in value and a 2% increase in quantity. South Africa’s average unit export value was USD 713 per ton, reflecting a competitive positioning. Other major exporters included Türkiye, the Netherlands, Mexico, and the United States, each with trade patterns reflecting regional proximity and specialisation.

**Figure 2:**
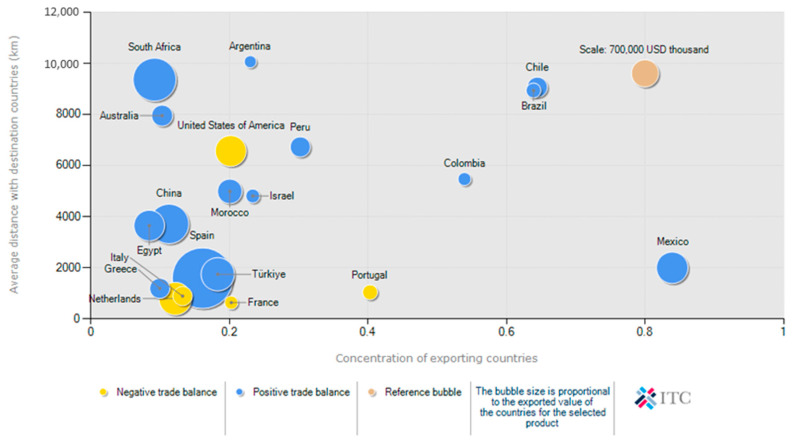
Concentration of exporting countries and average distance to their destination countries for the selected product in 2024. Sources: ITC calculations based on UN COMTRADE and ITC statistics.

This global export landscape showcases the diverse markets served by leading citrus-producing countries, with South Africa’s exports reaching destinations across Europe, Asia, and beyond. Figure 2 illustrates the geographical distribution of South Africa’s citrus exports in 2024, highlighting an average import distance of 9355 km and a market concentration index of 0.09. This figure underscores the importance of robust cold-chain logistics in maintaining quality over vast distances, thereby enhancing South Africa’s broad market reach and export resilience. The postharvest supply chain utilises advanced techniques to maintain fruit quality during long-distance transportation, thereby sustaining this export success. Refrigerated storage and shipping, monitored by environmental sensors tracking air temperature and humidity, are essential for maintaining freshness, firmness, and marketability [3]. Relative humidity levels of 90–95% are crucial in preventing moisture loss, which can lead to dehydration, internal dryness, and texture degradation [4]. Commercial cold storage facilities utilise precise ventilation and humidity control to maintain optimal moisture levels, reducing the risk of dehydration and microbial contamination [5]. In addition, proper humidity management during cold treatment prevents condensation, which can promote fungal infections and spoilage. Temperature is the most crucial factor influencing fruit quality deterioration, ripening rate, and shelf life [6]. A 10 °C reduction in fruit temperature typically doubles shelf-life by slowing metabolic and deteriorative processes [7]. Monitoring pulp temperature is crucial for determining precooling endpoints and assessing cargo quality during maritime transport [6].

### 1.2. Scope and Significance of This Review

Research has explored postharvest quality deterioration under assumed idealised cold chain conditions, using uniform parameters for temperature, ripeness, fruit size, and thermal properties. Several studies on postharvest logistics, including port operations and cold storage protocols, have identified inefficiencies in citrus supply chains [8,9,10,11]. While a small number of studies have compared shipment temperature profiles with arrival quality, their findings are typically weak or inconsistent due to limited sensor density, short observational periods, or non-standardised quality scoring. As a result, robust, multi-season, paired temperature–quality datasets capable of supporting reliable correlation analyses are still scarce, leaving a significant gap in understanding how real-world temperature variability affects citrus quality across export cold chains. Therefore, a critical gap persists in using historical temperature data to evaluate the real-world quality outcomes of citrus exports. Most studies rely on controlled experiments or computational simulations, which fail to capture the variability of transcontinental shipments, limiting their applicability to industry practices. This systematic literature review (SLR) addresses this gap by synthesising 35 peer-reviewed studies (2013–2025) to evaluate the extent of historical temperature data utilisation, assess methodological approaches, and identify barriers to robust quality correlations. The significance of this SLR lies in its potential to inform data-driven strategies to optimise cold chain logistics and reduce postharvest losses. By highlighting the underutilisation of historical data and the limitations of current methodologies, the review provides a foundation for developing centralised data systems and advanced monitoring technologies. The findings are relevant to industry stakeholders, policymakers, and researchers, and align with South Africa’s commitment to reduce food waste by 50% by 2030 [12].

### 1.3. Literature Review

The quality of fresh produce is determined by the 3T principle—time, temperature, and product tolerance. This principle highlights that food quality deteriorates over time, with temperature playing a crucial role [13]. Low temperatures also cause considerable chilling injury to the fruit rind, lowering the fruit’s marketability [5]. In addition, temperature also impacts moisture loss and the risk of condensation. Moisture loss is exacerbated at high temperatures, whereas at low temperatures, even minor fluctuations can lead to condensation. Temperature effects vary across citrus varieties, influencing external and internal fruit quality. While satsumas require higher storage temperatures, other varieties, such as navel oranges and Valencia oranges, can tolerate colder conditions. However, quality deterioration persists with prolonged exposure to chilling conditions. Satsuma fruits stored at 5.8 °C exhibited minimal weight loss (0.84%) and retained firmness better than those stored at room temperature (23 ± 2 °C), highlighting the effectiveness of controlled low-temperature storage [14]. Similarly, Valencia oranges stored at 2 °C showed increased peel damage, whereas those stored at 5 °C maintained firmness; however, they exhibited significant weight loss under low-humidity conditions [15].

Most fresh fruits retain their quality best near 0 °C, with spoilage doubling or tripling for every 10 °C increase; however, citrus fruits require careful handling due to their sensitivity to cold temperatures [16]. Extended cold storage alters citrus cell membrane fluidity and disrupts the structure of proteins such as tubulin, thereby compromising physiological integrity [4]. Freezing temperatures (below −1 °C) damage fruit tissue, while storage below 3–4 °C can cause chilling injuries, such as peel pitting or browning, in varieties like Valencia oranges or ‘Marsh’ grapefruit [4,17,18]. Studies confirm this is not uniform across citrus types [4,19]. Differences in cultivar, orchard, production area, and fruit maturity at harvest greatly influence citrus susceptibility to chilling injury (CI) [4]. While cultivar and orchard variability are important, regional weather variations in production areas play a more significant role in affecting CI susceptibility. The ‘Valencia’ oranges (especially Turkey) have the highest risk of CI during postharvest export cold chain, compared to mandarins and grapefruit, with grapefruit (Star Ruby) having the least susceptibility to CI [4]. Shipping at temperatures below 2 °C increases the risk of irreversible rind damage, such as pitting and browning, which renders the fruit unmarketable [5].

Effective cold chain management is therefore critical for optimising fruit quality, reducing deterioration, and extending shelf-life [20,21]. Despite advancements in refrigerated containers, maintaining consistent storage and transportation conditions remains challenging [21,22]. For example, the global maritime cold chain of citrus fruits experiences fluctuations in humidity and temperature from the orchard to the end consumer [20]. The implications of any deviations from the optimal temperature can be stark. For instance, airflow short-circuiting can occur through gaps between pallets and container walls, or at the container’s rear, allowing cold air to bypass the cargo rather than effectively cooling it [22]. Consequently, this leads to uneven cooling, with some areas cooling less efficiently, and fruits at the bottom of the container, exposed to cold delivery air, becoming susceptible to CI [22]. In addition, shipment-specific challenges include blocked vent holes, airflow short-circuiting, and heterogeneous airflow distribution, which exacerbate quality deterioration. Poor carton stacking intensifies cooling heterogeneity, contributing to rind pitting and mass loss in ‘Valencia’ oranges stored below 5 °C [23].

South African studies highlight logistical inefficiencies, such as port delays and congestion, contributing to these fluctuations [10,11,21]. A study at Cape Town Container Terminal found that 81% of refrigerated containers for summer fruits (grapes, plums, and pears) experienced temperature breaches above 2 °C, with 36% facing at least one breach and 22% failing to cool adequately before leaving the terminal [21]. Similarly, high-temperature spikes were observed at the start of the citrus fruit supply chain [11]. As a result, stakeholders are increasingly focusing on in-transit monitoring of temperature and humidity conditions. In commercial fresh produce export, governmental organisations such as the U.S. Department of Agriculture—Animal and Plant Health Inspection Service (USDA-APHIS) and the South African Perishable Products Export Control Board (PPECB) use fruit core or pulp temperature measurements to monitor the shipments [6,24]. These organisations use this data to determine whether the cargo complies with cold treatment protocols designed to kill specific phytosanitary organisms (e.g., fruit fly, false codling moth). Pulp temperature is generally monitored rather than air temperatures in commercial postharvest operations because it more accurately represents the fruit’s current thermal status and the excess heat that must be dissipated [25].

To monitor the temperature, standard equipment in refrigerated containers and precoolers includes wireless, self-powered data loggers with built-in sensors, such as iButtons^®^, which measure core temperatures [24]. In addition, smart sensor tags, often based on RFID technology, are employed to measure air temperatures inside the cargo [25]. However, these systems are limited by sparse deployment (3–5 sensors per 40 pallets), invasive installation, and reliance on air-temperature proxies, which fail to capture pulp-temperature heterogeneity [6,25]. The fruit pulp temperature lags behind instantaneous fluctuations in the supply air temperature, due to the thermal inertia of the fruit [25]. In addition, the limited use of sensors placed within a commercial shipment is linked to the high cost, the additional workload of placing and retrieving the sensors, and the time and expertise required to analyse the data [22]. Cheaper alternatives have been explored [24]. Cellular loggers (e.g., Sensitech TempTale^®^ GEO Eagle, Beverly, MA, USA) were reported to offer real-time visibility during land-based stages (e.g., pre-cooling, loading) and are recommended because they are easier to install and are cheaper ($40–$120 vs. $250–$300 for USDA probes). However, connectivity issues limit their effectiveness during the sea leg, leading to inconsistent recordings [24].

Beyond these individual container-level monitoring practices, temperature recording is also embedded within broader industry-level regulatory frameworks, providing a basis for understanding shipment-level thermal behaviour. Industry-level monitoring demonstrates that, beyond individual logger deployments, large volumes of temperature-related data are routinely generated within commercial citrus cold chain operations. In South Africa, the PPECB Handling Protocol (HPO1) requires vessels to be fitted with prescribed air (and preferably pulp) temperature sensors connected to calibrated recording systems (accuracy ±0.5 °C) [26]. Shipping lines are obligated to retain complete voyage temperature logs and submit them to PPECB upon request; failure to do so constitutes non-compliance and may result in liability for temperature-related quality losses. Furthermore, PPECB may compile technical reports on pre-shipment and voyage temperature conditions upon formal written request, typically linked to insurance claims [26]. In addition, industry data platforms such as Agrihub illustrate the increasing digitalisation of export logistics and shipment tracking. Despite this substantial operational data generation, temperature records are typically retained within proprietary reefer systems, limiting systematic access for research purposes. These provisions affirm that continuous temperature monitoring throughout the sea leg constitutes a recognised regulatory obligation and that comprehensive historical temperature records are maintained during standard commercial activities. However, access to such datasets remains conditional, case-specific, and driven by compliance requirements. Consequently, these records are not systematically consolidated into structured databases suitable for large-scale predictive temperature–quality modelling. Nonetheless, the existence of these operational records, although they are siloed, provides significant reference value for research. They highlight gaps in data accessibility and demonstrate potential applications for evaluating when and where quality losses occur, ultimately informing strategies to reduce variability and improve shipment outcomes.

A clearer understanding of when and where quality losses occur in the supply chain is critical for reducing variability within and between shipments, ultimately minimising food loss. Researchers have mapped temperature profiles and their impact on food quality; however, laboratory-scale experiments face significant limitations. Researchers have mapped temperature profiles and their effects on food quality; however, laboratory-scale experiments have significant limitations. These experiments often require days to cool cargo due to slow cooling processes, involve high workloads, and are resource-intensive. In contrast, full-scale experiments, typically conducted on commercial shipments, are performed only sporadically. Such experiments are costly, as the fruit required to fill a full container often has a market value of several thousand dollars and carries a substantial risk of partial loss [22,27,28]. To overcome these limitations, recent research has shifted toward computational approaches, leveraging physics-based simulations to rapidly evaluate different scenarios in silico. Computational fluid dynamics (CFD) models are increasingly used to optimise airflow within containers, improve stowing strategies, assess ventilated packaging, and simulate thermal quality degradation of produce [7,10,23,29,30,31]. For example, a Virtual Cold Chain (VCC) method using CFD was developed to track temperature-time histories in individual citrus fruits, effectively identifying airflow bottlenecks and regions prone to CI caused by vent-hole blockages [23]. This work was extended to implement a two-phase porous medium model (47% porosity, 487,803 finite elements) capable of simulating entire Navel orange containers (~102,400 fruits), achieving a balance between computational accuracy and efficiency [22].

More recently, digital twins (DTs) and related technologies have enhanced cold chain predictability for various fruits, including strawberries [32,33,34], oranges [3,4,20,35] and mangoes [36]. DTs in food supply chains utilise sensor data from logistics stages to simulate thermal and physiological changes, with a primary focus on modelling and predicting quality evolution [13]. Consequently, addressing challenges such as fluctuating environmental conditions, delays in accessing accurate temperature and humidity data, and imprecise shelf-life predictions can be achieved through real-time virtual representations of physical objects. A pre-harvest digital twin for ‘Turkey’ Valencia oranges was developed to model fruit growth across four South African regions (2018–2020) using the EPIC crop growth model and empirical regression models calibrated with literature and experimental data [35]. Coupled with weather data (temperature, humidity, radiation), the model predicted fruit quality parameters, revealing that warmer regions (e.g., Letsitele) produced larger, heavier fruit with balanced TSS/TA ratios (~10) compared to cooler regions (e.g., Sunday River Valley (SRV)). However, its reliance on literature-based calibration and omission of post-harvest dynamics limit its applicability in cold chains. In contrast, a digital replica was developed to model the impact of pre-harvest growing conditions on post-harvest quality for the same oranges [20]. It predicted similar regional trends (e.g., 8–12% mass loss in SRV), however, with lower accuracy (R^2^ ≥ 0.70) due to empirical transpiration rates and threshold-based CI modelling, making it less precise for cold chain optimisation. Combining a DT with a bidirectional LSTM model, reported temperature deviations of ±0.3 °C and shelf-life errors of ±1.2 days, reducing food loss by 8.6–15.5% using minimal sensors [13]. These examples demonstrate how emerging technologies, particularly, CFD-based simulations, DT, IoT sensing, and machine–learning–based quality models, identify new opportunities to uncover intangible quality losses, determine critical loss points, and provide technical assistance to enhance the efficiency of cold chain logistics.

Implementing advanced computational and analytical tools in the citrus cold chain involves clear trade-offs among modelling accuracy, infrastructure costs, interoperability, and operational feasibility. To facilitate technology selection in industrial contexts, these tools can be comparatively evaluated along four decision axes: modelling accuracy, implementation and operational cost, data dependency, and scenario suitability. CFDs provide exceptionally high spatial and temporal resolution, enabling the identification of hidden thermal “hot spots” and the calculation of individual fruit core temperature capabilities that are physically unattainable using discrete sensor networks [37]. However, CFD offers high physical fidelity; accuracy is constrained by necessary simplifications, including idealised geometries, wall-function turbulence treatments, and omission of buoyancy and radiative effects, which can lead to deviations from observed airflow and cooling patterns in ventilated packaging and palletised systems [31,38,39]. Heterogeneous heat transfer in packed beds further reduces predictive precision [23]. Secondly, high-resolution CFD necessitates millions of control volumes and multi-day runtimes, even for single-column or pallet-scale simulations, and frequently requires specialised hardware and expert configuration. Such computational demands prohibit real-time or routine operational deployment. Thirdly, CFD accuracy relies on detailed inputs; vent geometry, porosity, fruit thermal properties, and airflow resistances, which vary by cultivar, packaging design, and stacking arrangements. Incomplete or ambiguous data can lead to significant modelling inaccuracies, thereby requiring comprehensive calibration with experimental measurements [23,31,39]. Lastly, CFD is best suited for design optimisation, packaging evaluation, and DT scenario testing rather than time-critical cold chain decision-making. Its slow turnaround, sensitivity to assumptions, and dependence on controlled inputs limit its usefulness in dynamic commercial environments, such as in-transit monitoring or real-time container management [23,31,38,39].

Building on mechanistic CFD insights, DT and VCC extend modelling capacity into real-time virtual representations that enable closed-loop optimisation and dynamic quality control, with reported reductions in food loss of 8.6% to 15.5% [13]. More broadly, emerging technologies, including CFD simulations, DT architectures, IoT-enabled sensing, and machine-learning-based quality prediction models, demonstrate strong potential to uncover intangible quality losses, identify critical loss points, and enhance cold-chain decision support. Beyond postharvest logistics, DT integration in agriculture has expanded into precision crop management, livestock monitoring, and supply chain optimisation, including CNN-based disease detection supported by satellite imagery [40,41]. In comparison to CFD, DTs offer greater operational applicability through real-time synchronisation; however, this expanded functionality significantly increases system complexity, integration cost, and data-governance requirements.

CFD-based VCCs and physics-based DTs can simulate airflow distribution, thermal heterogeneity, and hygrothermal dynamics with high spatial granularity; however, their accuracy depends on continuous synchronisation with high-quality, high-volume data streams [41,42]. In practice, fragmented data ownership, sparsely instrumented shipments, and limited linkage between temperature records and arrival-quality assessments restrict the robustness of model calibration [43]. Predictive analytics and machine-learning regression models, such as XGBoost, demonstrate superior performance in generating fruit-acceptance scores and handling unseen interruptions [15,44]. However, their predictive reliability is dependent on large, balanced, and well-labelled training datasets, conditions seldom met in export environments characterised by inconsistent metadata standards and limited quality traceability. Accordingly, machine-learning models are most appropriate in environments where structured historical datasets and arrival-quality linkages are already institutionalised, rather than in fragmented supply chains lacking integrated data governance.

Consequently, DT implementation faces multi-dimensional barriers spanning technological, commercial, and human factors [41,42,45]. Technological challenges are the most significant obstacle to ensuring interoperability among heterogeneous systems and legacy equipment. Commercially, organisations face difficulties such as high and often unpredictable development costs, as well as the absence of clear methodologies for calculating long-term return on investment (ROI) and managing associated risks [45]. At the operational level, the prevailing commercial imperative for speed and throughput over scientific precision perpetuates minimal sensor deployment and fragmented data governance [8,22]. Therefore, technology selection in the citrus cold chain is not purely a question of modelling accuracy, but of strategic alignment between analytical depth, infrastructure readiness, data maturity, and commercial risk tolerance. Beyond the discussion of technological capability and industrial barriers, a more fundamental question persists: how has historical temperature data been used in the literature to model quality degradation, and to what extent have methodological approaches accounted for biological variability across the pre- and post-harvest continuum?

## 2. Materials and Methods

A Systematic Literature Review (SLR) was conducted to synthesise existing research on citrus cold chain management, focusing on the use of historical temperature data to understand quality degradation, the methodologies applied, and the extent to which pre- and post-harvest variability has been addressed. Unlike narrative reviews, which provide broad discussions without predefined selection criteria [46,47], an SLR follows a rigorous and structured approach that employs explicit inclusion and exclusion criteria to ensure transparency, reproducibility, and consistency [48]. This methodology enables researchers to systematically identify trends, uncover knowledge gaps, and evaluate the robustness of temperature-related quality assessments. Ultimately, this review aims to inform data-driven strategies for mitigating postharvest losses in global citrus supply chains, particularly those involving South African exports.

### 2.1. Research Questions Guiding the SLR

This review systematically investigates the extent to which historical temperature data have been available and used in citrus postharvest research, addressing the following key questions:

- How has postharvest quality deterioration in citrus been studied in the literature, particularly in relation to the role of temperature?
- To what extent have historical shipment and quality data been used to identify deterioration patterns, and what limitations have arisen due to data unavailability?
- How have past studies approached temperature-related quality assessments, and have they primarily relied on short-term experimental setups or simulations due to a lack of historical temperature records?
- Are there studies that correlate historical temperature fluctuations with fruit quality upon arrival, and what methodologies have been employed?

By addressing these questions, this study aims to demonstrate that prior research in the citrus cold-chain lacked access to comprehensive historical temperature and corresponding citrus quality data, necessitating the use of experimental or simulated temperature recordings, which prevented a definitive link between temperature deviations and quality issues in citrus. To overcome this limitation and support future empirical advancement in the field, this review developed a Minimum Common Data Model (MCDM) that specifies the metadata standards and quality-assessment metrics required to generate interoperable paired temperature–quality datasets. This model is derived inductively from the systematic synthesis of methodological, technological, and data-availability patterns identified in the literature, and is intended to provide actionable direction for enhancing data-driven decision-making in the South African citrus industry, improving supply chain efficiency, and reducing postharvest losses.

### 2.2. Data Search Strategy and Database Selection

The SLR in this study adheres to the PRISMA 2020 guidelines (Supplementary Materials), which provide a structured framework for transparency and reproducibility via a 27-item checklist and a four-stage flow diagram: Identification, Screening, Eligibility, and Inclusion. In parallel, the review process followed the three-phase model proposed by Kitchenham [49], planning, conducting, and reporting to ensure methodological rigour. During the planning phase, the need for this SLR was assessed by reviewing existing literature on citrus postharvest quality and evaluating the availability of temperature and quality datasets. Therefore, it justifies the focus on historical data gaps. In the conducting phase, a systematic search strategy was developed using key terms derived from the research questions. This stage included identifying records through database searches and screening titles and abstracts using predefined inclusion and exclusion criteria.

Full-text articles were then assessed for eligibility, with reasons for exclusions documented. The included studies were organised using EndNote version 21.1 (Build 17328), a reference management software package from Clarivate, Philadelphia, PA, USA, which facilitated the categorisation of citations and improved review accuracy through its grouping functionality. The reporting phase involved data extraction (see Section 2.3) and synthesis (see Section 3), where findings were analysed to highlight trends, knowledge gaps, and insights for data-driven improvements in the South African citrus sector. A total of 122 records were retrieved through structured database searches, as summarised in Table 1. The search queries were formulated using targeted keywords related to citrus, postharvest, temperature, and export supply chains, in alignment with the study’s research objectives.

### 2.3. Study Selection and Screening Criteria

The selected studies adhered to a rigorous methodology, applying explicit inclusion and exclusion criteria to ensure alignment with the research. Only original research articles were included across all databases to ensure a focused and relevant literature base, while review articles, book chapters, and conference papers were excluded. Titles and abstracts were initially screened for explicit use of actual shipment temperature data, prioritising historical trends over theoretical or lab-based findings. However, given the idiosyncrasies of SCM research [50]. A deeper examination was necessary to assess relevance beyond standard metadata. This approach acknowledges that abstracts often provide broad information; however, they seldom reveal the depth of construct definitions, operationalisation, or data embodiment, necessitating further scrutiny for alignment with the SLR’s objectives.

For the studies to be included, the selection of studies had to adhere to these criteria:

- Language restrictions: Only studies published in English were included to ensure accessibility and consistency in analysis.
- Publication Criteria: Peer-reviewed journal articles published between 2013 and 2025 were considered for inclusion.
- Research Focus: Studies on citrus postharvest quality deterioration in export supply chains, emphasising temperature-related impacts.
- Data and Methodology:
  - Empirical studies incorporating shipment temperature data, whether primary, secondary, experimental, or simulation-based, to account for the limited availability of historical records.
  - Studies with validated data collection techniques, clear operationalisation of variables, and robust statistical analyses ensuring methodological reliability.
  - Studies that explicitly compared experimental temperature setups with actual shipment records, allowing for an assessment of real-world versus controlled conditions.
- Real-World vs. Controlled Environments: Studies using actual shipment records were prioritised. However, simulation-based studies were included when historical temperature data were unavailable, recognising the field’s reliance on experimental setups.

Studies were excluded if they met any of the following conditions:

- Language Restriction: Articles not written in English were excluded.
- Conference papers, review articles, and book chapters were excluded to maintain methodological rigour, though this may limit insights from industry reports.
- Pre-Harvest Focus: Research addressing pre-harvest factors unrelated to post-harvest temperature fluctuations.
- Commodity and Supply Chain Scope: Studies on non-citrus commodities or unrelated supply chains were not considered.
- Shipment Temperature Data Screening: Titles and abstracts were screened to focus explicitly on actual shipment temperature data, prioritising historical trends over theoretical or lab-based findings. Studies without shipment data were categorised separately for gap analysis to assess the field’s reliance on controlled environments versus real-world shipment conditions.

**Figure 3:**
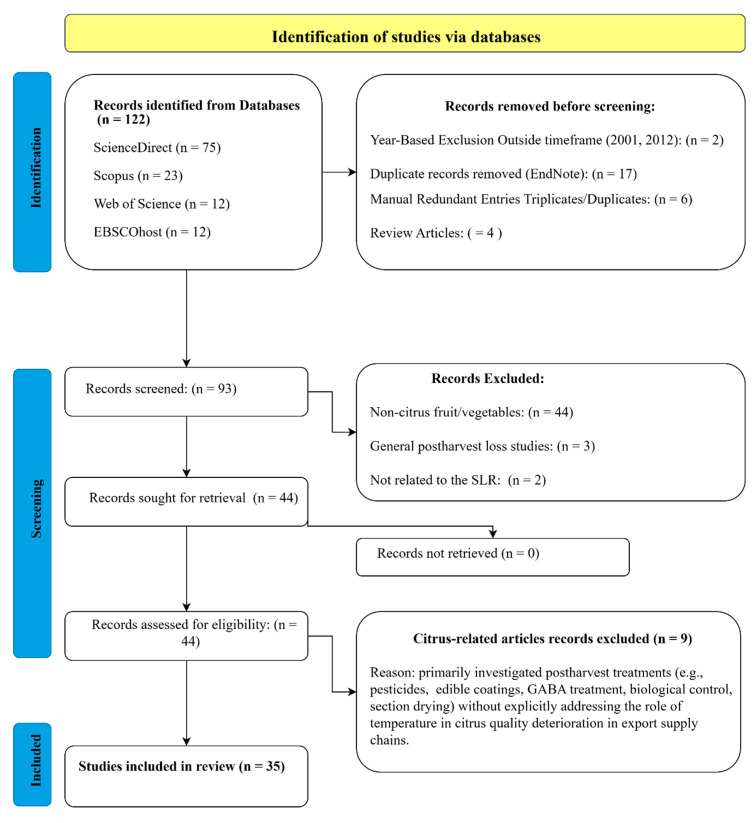
The PRISMA flow diagram illustrates the Identification, Screening, and Inclusion of studies on the role of temperature in the deterioration of citrus quality within export supply chains.

Figure 3 illustrates the results of the systematic review process, detailing the identification, screening, and inclusion of studies focused on the role of temperature in citrus quality within export supply chains. A total of 122 records were initially retrieved from four databases, as detailed in Table 1. After removing 23 records due to duplicates, timeframe restrictions, and review articles, 93 records proceeded to the screening phase. Of these, 49 were excluded because they did not align with the SLR’s core focus, such as studies unrelated to citrus or those addressing general postharvest loss trends without a specific reference to temperature. The remaining 44 full-text articles were assessed for eligibility. During this stage, nine studies were excluded because they did not address temperature-related postharvest quality deterioration. For example, Ref. [51] focused on yield prediction using weather and heat units; however, it did not examine quality deterioration during export. Other studies, for example, Refs. [52,53,54,55,56,57] investigated non-temperature-specific interventions such as pesticide degradation, edible coatings, GABA treatment, biological control, or section drying, and therefore, fell outside the scope of this review. Studies such as [57], which examined the effects of fruit size under controlled storage at 10 ± 0.5 °C for 150 days, provide insights into the long-term impacts of storage on citrus quality. However, their focus on size rather than temperature variability or real-world shipment conditions limits their applicability. Ultimately, 35 studies met the inclusion criteria and were incorporated into the final review, all of which directly addressed the influence of temperature on citrus quality within the context of export supply chains.

### 2.4. Data Analysis Approach

This section outlines the analytical methods employed to address the review objectives. A two-pronged approach was adopted. First, bibliometric mapping was used to visualise research trends and identify knowledge gaps in the existing literature. This analysis aims to investigate the contributions of various nations to the adoption and development of post-harvest technology, emphasising key players, emerging trends, and opportunities for international collaboration that could accelerate industry transformation. Second, structured data extraction was conducted to systematically gather and synthesise evidence in line with the review questions. These complementary methods provided both a high-level overview of thematic developments and a detailed analysis of individual study contributions.

#### Bibliometric Mapping (VOSviewer^®^)

To identify research trends and knowledge gaps in postharvest citrus studies, bibliometric mapping was conducted using VOSviewer^®^ version 1.6.20 on a dataset comprising 35 peer-reviewed journal articles selected for the SLR. This tool applies a “visualisation of similarities” technique, positioning terms based on their co-occurrence relationships to detect clusters at multiple resolution levels [58,59]. To identify and select terms for VOSviewer^®^’s bibliometric mapping, text data (in English only) undergoes five steps: copyright statements are removed if selected, sentences are detected using Apache OpenNLP, words are tagged by part of speech, noun phrases are identified as sequences ending in a noun with adjectives or nouns, and phrases are standardised by removing non-alphanumeric characters, accents, converting to lowercase, and singularising plurals [60].

VOSviewer^®^ employs text mining to generate a two-dimensional co-occurrence network map of keywords, revealing emerging research areas, trending topics, and the historical evolution of scientific fields. Co-occurrence analysis evaluates word relationships by measuring the frequency with which keywords appear together in these documents; keywords are deemed to co-occur when they appear simultaneously in the same context. On the map, each keyword is represented as a circle, with its size indicating the number of publications that contain the keyword [61]. Keywords positioned closer together co-occur more frequently, with their distance reflecting relatedness based on co-occurrence frequency, while curved lines highlight strong relationships [61,62,63]. The clustering approach groups related keywords into colour-coded clusters, enabling researchers to uncover dominant themes and identify underrepresented areas, as the spatial arrangement of keywords reflects their conceptual relatedness [63,64].

To investigate the bibliometric structure of the literature, a dataset from an SLR was analysed using VOSviewer^®^, with data sourced from supported databases (Scopus and Web of Science) and supplemented by manually retrieving articles from unsupported databases, such as ScienceDirect and EBSCOhost (Academic Search Premier), via Scopus where available, and integrating them into a consolidated CSV file. Three bibliometric analyses were conducted using VOSviewer^®^’s full counting method to ensure equal weighting of occurrences. The first analysis examined co-authorship among countries, treating them as the unit of analysis, to map patterns of international collaboration. The second analysis focused on co-authorship, using individual authors as the unit of analysis, to identify key contributors and their collaborative networks. The third analysis used the co-occurrence of author keywords to uncover dominant research themes and conceptual linkages.

### 2.5. Data Extraction for SLR Questions

Data extraction followed a predefined extraction form to ensure consistency and alignment with the review objectives [49]. The form was designed to capture key elements necessary to answer the SLR questions, including study methodologies, shipment temperature records, treatment effects, and confidence intervals. These data fields enabled effective summarisation and comparison across studies, which is important for synthesising patterns in postharvest citrus deterioration. Additional fields captured constructs, operational definitions, and theoretical frameworks to support methodological rigour. All extracted data were systematically organised and presented in integrated tables for clarity, as outlined in Table 2. Microsoft Excel^®^ facilitated the initial structuring.

## 3. Results and Discussion

The synthesis of 35 peer-reviewed studies investigating the impact of temperature on postharvest quality deterioration in citrus fruits is presented. The first part provides a visual and thematic overview of the existing literature, while the second part offers an in-depth synthesis aligned with the SLR questions.

### 3.1. VOSviewer^®^ Co-Occurrence Mapping Insights

This section presents the results of a bibliometric analysis using VOSviewer^®^ to map keyword co-occurrence networks and identify research trends, thematic clusters, and knowledge gaps within the selected literature.

### 3.2. Co-Authorship Analysis by Country

To assess the global distribution of publications within the 35 peer-reviewed journal articles included in the systematic literature review, a co-authorship analysis was conducted using countries as the unit of analysis. A minimum threshold of three documents per country was applied, resulting in six countries meeting the inclusion criteria out of a total of 17. For each of these countries, the total link strength, representing the total strength of co-authorship connections with other countries, was calculated [60]. The countries with the highest total link strength were South Africa (2860), Switzerland (2693), and Belgium (1689), indicating robust international collaboration networks. In terms of publication volume, South Africa led with 17 documents, followed by Switzerland with 14 and Belgium with 6. In addition, two distinct clusters of countries were identified based on co-authorship patterns. Cluster 1 included China, the Netherlands, and the United States, while Cluster 2 comprised Belgium, South Africa, and Switzerland. These clusters reflect regional and collaborative research dynamics among the top contributors to postharvest citrus research.

### 3.3. Co-Authorship Analysis by Author

The second analysis employed co-authorship, using individual authors as the unit of analysis to explore collaboration patterns among researchers. The analysis was conducted on the 35 peer-reviewed journal articles included in the systematic literature review. To ensure consistency, first names were reduced to initials, and a minimum of 2 documents per author was required. Out of 121 authors, 29 met this criterion. During the co-authorship analysis, VOSviewer^®^ identified that not all authors were connected within a single collaborative network. Of the 28 authors who met the inclusion criteria, only 16 formed a connected component, indicating active collaboration among them. The remaining authors were unconnected, suggesting limited or no co-authorship links with others in the dataset. For clarity and interpretability, only the largest connected set of 16 authors was visualised and analysed further.

**Figure 4:**
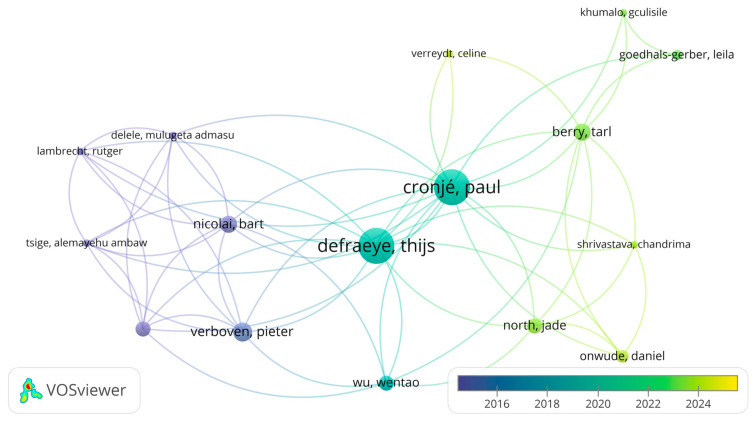
Co-authorship overlay visualisation map illustrating collaborative linkages among leading researchers in citrus postharvest management. A higher node density indicates that central figures contribute significantly to knowledge development within the field.

The top five most prolific authors, based on the number of documents, were Cronjé, P, and Defraeye, T, each with 17 publications and 705 and 773 citations, respectively. Followed by Verboven, P, with seven publications and 564 citations. Berry, T., and Nicolai, B. had six publications and 56 and 518 citations, respectively. Despite Berry T. having six documents, other authors, such as Opara UL with 370 citations and Delele M.A. with 240 citations, demonstrated greater citation impact based on the documents utilised in the SLR. It is important to note that these citation counts reflect only the citations received within the 35 peer-reviewed journal articles included in the SLR and do not represent the authors’ total citation profiles across all their published works. As shown in Figure 4, the co-authorship overlay visualisation generated by VOSviewer^®^ highlights the interconnectedness of these leading scholars.

Notably, Cronjé, P, and Defraeye, T, stand out as central figures, distinguished by their high publication volume and extensive collaborative networks, with all 15 of their 17 papers involving co-authorship. These 15 papers collectively advance the understanding of citrus postharvest management through the innovative use of digital twins, CFD, and integrated assessments [3,5,20,22,35,37]. They highlight the critical roles of cooling strategies and ambient loading [16,57,58], packaging design [8,58,59], and pre-harvest variability in determining postharvest fruit quality [4].

North, J. and Berry, T., have also made significant contributions by investigating the influence of pre-harvest variability on postharvest quality [3,20,35]. Research on cold chain logistics, exemplified by [65], has progressed in tandem, while [23] has facilitated linkages between research clusters through pioneering studies on virtual cold chains. Collectively, these research efforts underscore a transformative shift in citrus postharvest management toward data-driven, digitally optimised, and sustainability-focused supply chains.

### 3.4. Co-Occurrence Analysis of Author Keywords

The third analysis focused on co-occurrence, using author keywords as the unit of analysis to uncover dominant research themes and conceptual linkages within the field of postharvest citrus studies. This co-occurrence technique served as a pivotal tool for deciphering patterns in the corpus of 35 peer-reviewed journal articles on postharvest citrus research, enabling the identification of five distinct clusters [66]. From the SLR articles, 123 unique keywords were initially extracted. However, after using the thesaurus file to standardise variations in keyword terminology, the total is 80 keywords. A minimum threshold of two occurrences was applied, yielding 31 keywords. After excluding three unrelated keywords, 28 remained for analysis.

**Figure 5:**
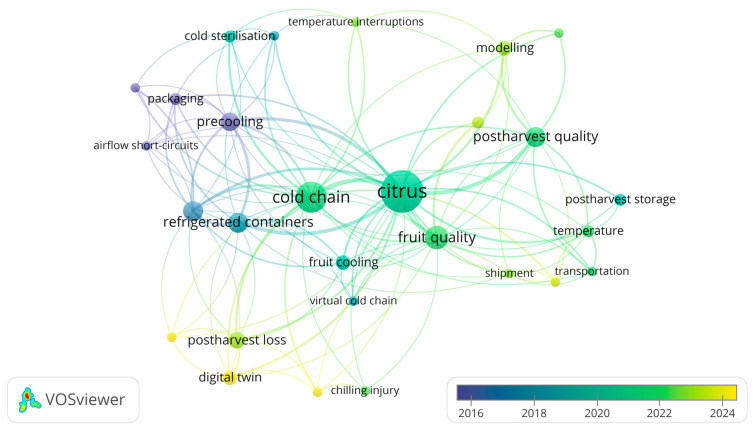
Keyword co-occurrence network generated using VOSviewer^®^. An overlay visualisation illustrating thematic and methodological clusters across postharvest citrus research. Node size indicates keyword occurrences, distance reflects relatedness, and colours denote the temporal and methodological groupings.

**Figure 6:**
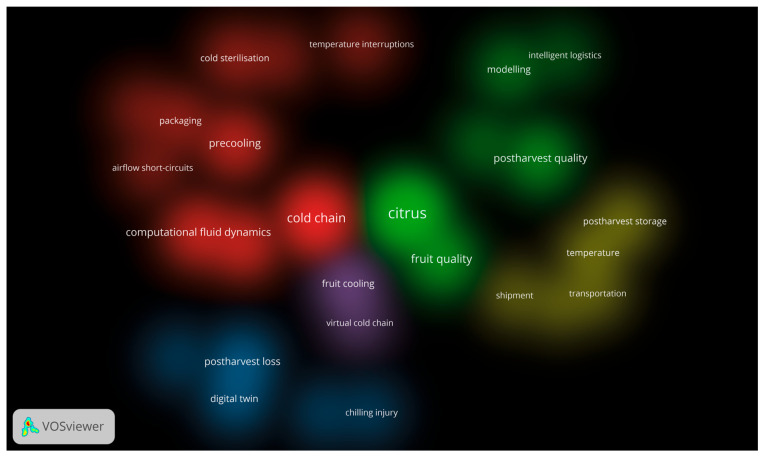
Density visualisation of keyword co-occurrence generated using VOSviewer^®^, highlighting key research themes in postharvest citrus studies. Colour intensity reflects keyword density, with “citrus” and “cold chain” as central themes.

These 28 keywords were then analysed to identify co-occurrence patterns, which served as the basis for clustering. Each cluster, characterised by a unique set of keywords with similar co-occurrence patterns, was visually represented in VOSviewer^®^. The overlay visualisation (Figure 5) positions ‘citrus’ and ‘cold chain’ as central nodes with high connectivity, with a temporal gradient from 2016 (blue) to 2024 (yellow) showing the evolution of research focus. The density visualisation (Figure 6) highlights areas of high co-occurrence density, with ‘citrus’ and ‘cold chain’ in the brightest green and red regions, respectively, confirming their prominence. These clusters and visualisations illustrate an evolution in citrus postharvest research from foundational logistics and quality studies (2016–2020) to advanced, data-driven, and sustainability-focused approaches (2020–2024). The high-density regions around ‘citrus’ and ‘cold chain’ (Figure 6) reinforce their role as core research pillars. Meanwhile, emerging themes such as ‘digital twin’ and ‘virtual cold chain’ (yellow regions in Figure 5 and Figure 6) highlight gaps in the literature, offering opportunities for new research, such as integrating digital technologies with traditional cold chain practices to enhance efficiency and reduce postharvest losses [64].

### 3.5. Synthesis of SLR Findings

This section presents a systematic synthesis of 35 peer-reviewed studies investigating the impact of temperature on postharvest quality deterioration in citrus fruits. Before addressing the SLR research questions, it is essential to distinguish the methodological evidence streams represented in the corpus. The 35 included studies fall into three distinct categories: (i) real-world shipment data, capturing operational temperature behaviour in commercial supply chains; (ii) controlled experimental studies, which isolate physiological responses to temperature under idealised conditions; and (iii) simulation-based approaches, including CFD, virtual cold chains, and digital twins that model airflow, heat transfer, and quality evolution. Table 3 summarises these streams, identifies which studies adopt each approach, and outlines the explicit variables, quality attributes, and temperature-related outcomes investigated by each study. This categorisation clarifies that, while experimental and simulated studies dominate the field, only a small subset uses actual shipment records, and none combine temperature and quality data across multiple seasons, thereby establishing the empirical gaps addressed in the SLR findings that follow.

I. How has postharvest quality deterioration in citrus been studied in the literature, particularly concerning the role of temperature?

Postharvest citrus quality deterioration has been investigated using three distinct methodological evidence streams: (i) studies using real-world shipment temperature data, (ii) controlled experimental studies and (iii) Computational simulation-based analyses (CFD, VCC, DT). Studies typically focus on optimising cooling processes (e.g., precooling, ambient loading) and storage conditions to minimise quality losses during export supply chains. A variety of quality parameters have been employed to assess deterioration, including weight loss, electrolyte leakage, CI, decay rate, sensory attributes and physicochemical properties.

Two articles [24,79] were not included in the synthesis for Question 1 of the SLR, as neither directly addresses how temperature influences postharvest quality deterioration in citrus. The synthesised literature is divided into three approaches: experimental trials, computational simulations, and real-world shipment-based monitoring. The remaining 35 studies fall into the three methodological categories described below.

The first category is the Real-World Shipment studies. This evidence stream monitors authentic transcontinental and regional citrus shipments to reveal where logistics and temperature control succeed or fail under actual commercial conditions. Representative studies include [3,5,8,65,67,68,69]. Using time–temperature records from 43 export shipments and physics-driven digital-twin modelling, researchers showed that hygrothermal variability between shipments drove substantial differences in end quality at retail, the fruit quality index (FQI) varied by >20% (≈20–43%) and remaining shelf life (RSL) differed by 3 days across shipments [3]. Therefore, resulting in a 3-day spread in remaining shelf life and a 20% variation in the fruit quality index upon arrival at retail. Commercial container experiments instrumented multiple pallets and layers to quantify cooling rates, hot spots, and variability during 18-day marine transport [69]. Results linked loading temperature and uncontrolled airflow gaps/short-circuits to heterogeneous cooling and highlighted the benefit of partial precooling (≈10 °C) for reducing hot spots and chilling-injury risk.

Controlled experimental studies assess the effects of fixed or intentionally manipulated temperature regimes and storage conditions to isolate mechanisms of physiological deterioration [4,14,15,43,44,70,71,72,73,74,75,76]. For example, Ref. [14] evaluated the difference in Satsuma quality after storage at low temperature (5.8 °C) and room temperature (23 ± 2 °C) for 60 days, showing accelerated weight loss and firmness decline at warm temperatures. Some studies use experimental data to develop and evaluate regression models for predicting fruit acceptance scores based on pre-harvest and postharvest factors [15,43,44,70]. These studies provide rigorous physiological evidence; however, they lack real-world thermal variability.

Computational simulations include CFD, VCC, and DT [3,5,7,13,20,22,23,27,35,37,38,39,77]. For example, Refs. [39,77] combined physical forced-air cooling experiments with high-resolution CFD modelling to evaluate citrus cooling performance under different package designs. In the 2013 study, experiments on pallet-stacked ‘Valencia late’ oranges with in-fruit temperature monitoring were used to validate a discrete-fruit CFD model that resolved airflow and heat transfer around individual oranges. The 2014 follow-up study extended this approach by using CFD exclusively to analyse container airflow resistance, convective heat-transfer coefficients, cooling rates (HCT, SECT), and the associated energy requirements for forced-air cooling, providing detailed mechanistic insight into how packaging design and operating conditions influence temperature management in the citrus cold chain.

In addition to mechanistic CFD and DT simulations, several studies employ statistical modelling and macro-logistical optimisation frameworks to analyse citrus postharvest performance at the supply-chain level. These studies use predictive statistical relationships, machine-learning models, and logistics-oriented optimisation approaches to evaluate quality outcomes, storage behaviour, or system-level decision-making [43,44,70,78]. For example, Ref. [78] designed a mixed-integer linear programming (MILP) bi-objective optimisation model to minimise total costs and carbon footprints in the inbound logistics of fresh Valencia oranges. In comparison, Ref. [43] uses machine-learning regression models (specifically XGBoost) to develop dynamic quality-prediction models that consider temperature interruptions in real time to support First Expired, First Out (FEFO) logistics. Large-scale phenotyping work, such as Owoyemi and Porat [44], uses machine learning to generate shelf-life prediction models from thousands of experimental data points, thereby enabling FEFO-style inventory decisions and batch-specific storage recommendations. Collectively, these studies support decision-making at the operational and logistical levels, offering predictive capabilities and optimisation insights that complement, however, do not replicate, the mechanistic detail of CFD or DT approaches.

II. To what extent have historical shipment and quality data been used to identify deterioration patterns, and what limitations have arisen due to data unavailability?

Historical shipment and quality data have been used to identify critical temperature breaks, calibrate digital twins, and develop predictive quality models, though researchers face persistent challenges due to limited sensor density and data loss [5,8,27,65,67,68,69]. In a study of 47 commercial citrus shipments from South Africa, air temperature sensors installed near the fruit were used to drive a mechanistic model that simulated heat transfer, quality decay, and the efficacy of cold disinfestation [5]. Similarly, temperature data from 43 shipments were used to build digital twins that quantified how hygrothermal variability between voyages causes significant fluctuations in end quality, such as a 3-day spread in remaining shelf life [3]. Similarly, six containers with varying initial fruit temperatures were used; however, the quality was not assessed [69].

Despite these advancements, many studies lack direct quality data, such as measurements of chilling injury, decay, or sensory attributes, relying instead on temperature as a proxy for potential deterioration. For example, research tracking pulp and ambient temperatures in 19 containers revealed significant onshore inefficiencies during pre-cooling and loading, which directly caused cold chain breaches [65]. Another study involving six containers linked loading temperatures and uncontrolled airflow gaps to heterogeneous cooling, although it noted that direct quality was not extensively assessed [69]. Further research identified specific areas along the South African export leg where temperatures exceeded prescribed ranges by conducting trials on two consignments of clementines and two consignments of navels [8]. Lastly, Ref. [68] tracked 30 shipments from Greece to Switzerland, identifying up to 10 °C variations, however, without quantifying quality impacts.

Current limitations in the availability and representativeness of temperature data significantly constrain cold chain diagnostics and quality modelling. In commercial practice, shipments typically deploy only three sensors per container, which masks intra-container thermal variability and cooling heterogeneity among individual fruits within the cargo [22]. Moreover, sensors are frequently positioned in easily accessible locations, such as near the container door, which may not accurately capture spatial gradients or reflect the overall evolution of fruit quality across pallet positions and vertical layers [37]. Historical shipment records further compound this limitation by monitoring ambient air temperature rather than fruit pulp temperature, despite pulp temperature being the critical metric for estimating physiological quality evolution and verifying compliance with phytosanitary and cold sterilisation protocols [22].

In-transit visibility remains another structural constraint. During the sea leg, exporters are generally unable to monitor temperature conditions in real time, as conventional data loggers require physical retrieval at destination ports, while cellular-enabled devices lose connectivity offshore [24]. High-throughput analyses are additionally undermined by low device retrieval rates and occasional logger malfunction, particularly at busy destination terminals [8]. Data governance issues also limit transparency: much industry-level research is proprietary, often manufacturer-driven, and temperature data collected during transit is frequently the property of shipping lines rather than fruit exporters [24,67]. Finally, physics-based and predictive quality models are limited by missing physiological and operational inputs, including imperfect pallet stacking, airflow obstruction, handling-related human error, and the initial fungal load of consignments [22]. These factors are rarely recorded in historical shipment logs; however, they materially influence real-world cold chain performance.

III. How have past studies approached temperature-related quality assessments, and have they primarily relied on short-term experimental setups or simulations due to a lack of historical temperature records?

Past studies have approached temperature-related quality assessments through three primary methodologies: controlled laboratory experiments, physics-based simulations, and real-world shipment monitoring. Because high-resolution historical temperature records from commercial transit are often sparse or unavailable, researchers have frequently relied on short-term experimental setups or computational models to infer quality degradation patterns. Controlled experimental approaches have been widely used, with at least ten studies employing controlled experimental designs to assess temperature-related degradation of citrus quality, focusing on physicochemical, sensory, physiological, and microbial attributes under specific temperature conditions. ‘W. Murcott Afourer’ mandarins were evaluated at 5 °C and 20 °C, using sensory panels and gas chromatography-mass spectrometry (GC-MS) to link aroma volatile changes to flavour loss [70]. Physicochemical and physiological traits have also been evaluated, with large-scale phenotyping of 12,000 ‘Rustenburg’ navel oranges and 10,800 ‘Valencia’ oranges providing foundational data linking storage time and temperature to quality acceptance scores, weight loss, and firmness [15,44]. Some studies specifically isolate triggers for chilling injury (CI) and decay, often using step-down cooling protocols to evaluate fruit tolerance [27].

Due to the high cost, labour intensity, and visibility gap of full-scale commercial trials, many researchers have relied on computational simulations. At least six studies modelled cooling rates and quality evolution in oranges, using CFD to assess package designs and cold-chain scenarios in the absence of historical data [7,26,36,37,75,76]. A notable advancement is the “Virtual Container” model, which was validated against full-scale experimental datasets to simulate the cooling of 102,400 oranges and identify the slowest-cooling hotspots that are physically difficult to monitor [22]. More recently, experimentally verified temperature data have been integrated with digital twin simulations and deep learning approaches (e.g., BiLSTM) to establish dynamic shelf-life systems capable of adapting to real-time fluctuations [13]. Similarly, Ref. [26] presents a dual methodology that reflects the broader trend of temperature-related assessments constrained by limited historical records. Cooling times were first estimated using computational simulations based on container refrigeration capacity, with the required power modelled as a function of the seven-eighths cooling time (SECT) and fruit thermal properties. These predictions were subsequently validated in a 22-day container experiment using pulp temperature sensors, which revealed that packaging and stacking often limit heat removal. This study demonstrates how combining short-term experimental setups with simulations can bridge gaps in sparse historical temperature data while validating predictive models.

Approximately eight studies incorporated real-time monitoring of commercial shipments [3,5,8,24,65,67,68,69]. However, these efforts have historically been limited. Data resolution is low, with only a few sensors (often three or fewer) per shipment, which masks thermal variability and makes it difficult to predict the quality of the entire cargo. Monitoring studies have primarily focused on detecting temperature breaks (deviations of 2 °C or more for 90 min) rather than performing comprehensive physiological quality assessments [8]. Nevertheless, some historical records have been successfully repurposed; for example, data from 47 shipments have been used to drive mechanistic models predicting pest mortality and chilling injury risk [5].

IV. Are there studies that correlate historical temperature fluctuations with fruit quality upon arrival, and what methodologies have been employed?

None of the 35 studies directly correlates longitudinal temperature fluctuations (as recorded in multi-season or multi-year postharvest shipment records) with the quality of citrus fruit upon arrival. However, multiple recent studies have explicitly addressed the correlation between shipment-specific temperature fluctuations and fruit quality upon arrival, using a range of methodologies from physics-based simulations to statistical and AI-driven models [13,22,37,43,70]. The examples include studies that developed physics-based digital twins of refrigerated containers and pallets using CFD and porous media models [7,22,37]. These models simulate airflow, heat transfer, and fruit cooling at high spatial resolution (e.g., 60,000 virtual probes), enabling prediction of temperature-driven quality loss. Recently, digital twins with deep learning (BiLSTM) have been used to dynamically predict in-box fruit temperature and shelf life [13].

In the study [70], predictive models were developed and validated using experimental data spanning several growing seasons. Internal quality traits (TSS, TA) were modelled from data collected during the 2007/2008 and 2008/2009 seasons in Egypt, while physical and pathological traits (weight loss, decay, and CI) were modelled from data collected during the 2004/2005 and 2005/2006 seasons in Florida. The study employed “thermal time” (accumulated degree days above or below specific thresholds) to quantify how cumulative temperature exposure over these periods directly determines the percentage of decay, CI, and weight loss. The models achieved high accuracy, with Nash-Sutcliffe Efficiency (NSE) values of 0.991 for CI and 0.965 for TA relative to observed values from individual years. While [3] the authors analysed original sensor data from a large longitudinal dataset of commercial shipments. The research utilised original air-temperature data from 43 transcontinental shipments of ‘Valencia’ oranges from South Africa to Europe, spanning the entire export season from August 2018 to September 2019. These historical records were linked to physics-based digital twins to quantify how hygrothermal variability between these 43 shipments affected quality metrics upon arrival. The findings revealed that fluctuations between shipments caused a 3-day spread in remaining shelf life and a 20% variation in the fruit quality index (FQI) at retail. In a separate study, data collected across the 2018/2019 and 2019/2020 production seasons from four regions (Citrusdal, Nelspruit, Letsitele, and Sunday’s River Valley) were used to assess chilling injury susceptibility over two consecutive production years [4]. By correlating regional weather records (including minimum temperatures, heat units, and vapour pressure deficit) from these two years with postharvest storage trials, the study identified that regional weather differences impact CI susceptibility more than orchard-to-orchard variability within a single season. Lastly, inter-shipment variability was monitored by measuring air and fruit core temperatures across 30 shipments from Greece to Switzerland, with the first 15 shipments monitored at the end of 2022 and the second batch in 2023 [68]. This original dataset enabled a quantitative comparison of inter-shipment variability, revealing average temperature differences of up to 9 °C between separate shipments and demonstrating that every shipment has a unique thermal history that dictates arrival quality.

### 3.6. Comparative Evaluation of Emerging Technologies

To provide a structured comparison of emerging technologies in the citrus cold chain, a Literature Synthesis Matrix was developed Table 4. The assessment aligns with the five predefined research questions that guide this systematic literature review (SLR): temperature-related deterioration (SLR Question I), use of historical data (SLR Question II), experimental versus simulated studies (SLR Question III), temperature–quality linkage (SLR Question IV), and data requirements and implementation considerations (SLR Question V). Each technological aspect is scrutinised across these dimensions to elucidate methodological focus, data prerequisites, and potential industrial barriers. This comparative overview identifies research gaps and provides a practical framework for selecting appropriate technologies based on accuracy, cost, and suitability for commercial cold chain operations.

### 3.7. Minimum Common Data Model

To operationalise the research gap identified in this review, specifically the absence of paired, longitudinal datasets linking in-transit temperature histories with destination quality, we propose a Minimum Common Data Model for citrus export cold chains. This framework standardises the required metadata, monitoring protocols, and quality rubrics to ensure that future datasets are interoperable and suitable for meta-analysis. The proposed schema is stratified into three echelons of data collection:

I. Initial Biological and Environmental Baseline

Preharvest climatic variability is a primary determinant of the development of postharvest disorders and CI susceptibility. Weather conditions after fruit set can induce physiological rind disorders and increase vulnerability to decay, particularly following rainfall events that promote oxidative stress and impaired gas exchange in flavedo tissues [80]. Quantitative evidence further demonstrates that regional weather variability significantly alters CI susceptibility: fruit from temperate coastal regions shows substantially higher risk than that from hotter inland production areas, and early-harvested fruit exhibits reduced cold tolerance [4,20,35]. Mandatory environmental fields (anthesis to harvest):

- Daily minimum and maximum temperature (°C).
- Rainfall (mm).
- Solar radiation (MJ m^−2^).
- Vapour Pressure Deficit (VPD, kPa).

Postharvest behaviour is path-dependent; therefore, datasets must capture the consignment’s initial biological and environmental state. Mandatory biological fields:

- Cultivar.
- Fruit diameter (FD, mm).
- Fruit weight (FW, g).
- Rind thickness (RT, mm).
- Initial Total Soluble Solids (TSS, °Brix).
- Initial Titratable Acidity (TA, %).

II. In-Transit Monitoring Schema

Existing phytosanitary cold-treatment protocols already define the minimum admissible monitoring configuration and can therefore serve as the regulatory baseline for the MCDM. This includes three pulp sensors placed in fixed, georeferenced positions, two air sensors measuring delivery and return air temperatures, and a sampling interval of no more than 60 min [26,81,82,83,84,85,86]. These parameters constitute the minimum temperature history required for inclusion in paired temperature–quality datasets.

For modelling and optimisation applications, an enhanced research layer is recommended. This includes increasing the sampling interval to ≤15 min, recording sensor position metadata (e.g., pallet row, stack height, and carton type), and logging relative humidity alongside temperature. In addition, real-time data transmission via 3G/4G-enabled loggers is recommended to ensure data continuity and immediate availability. Higher temporal resolution and structured spatial metadata substantially improve the capacity to resolve intra-container heterogeneity and strengthen downstream temperature–quality correlations.

III. Arrival and Outcome Data (The Quality Endpoints)

To enable meaningful temperature–quality correlation, datasets must terminate in standardised, objective arrival endpoints. A unified ordinal scale (e.g., 1–5 overall acceptance) must be applied consistently across studies to ensure comparability. Chilling injury indices, decay incidence, rind pitting severity, and firmness loss should follow predefined categorical definitions. Furthermore, mandatory physicochemical endpoints should include mass loss (% relative to initial fresh weight), final total soluble solids (TSS, °Brix), and final titratable acidity (TA, %). To ensure operational relevance, the dataset should culminate in an actionable metric: Predicted Remaining Shelf Life (RSL), expressed in days and defined as the duration required for the respiration-driven quality indicator to fall below the 10% marketability threshold. By integrating these structured fields, the framework enables Physics-Based DT to repurpose limited in-transit sensor data into comprehensive temperature–quality datasets suitable for modelling, benchmarking, and decision-making.

## 4. Limitations and Bias

Firstly, the review was restricted to studies published in English. This exclusion introduces potential language bias, as pertinent research published in other languages, particularly from major citrus-producing regions such as China, Brazil, and Spain, may not have been captured. Consequently, the findings may not fully represent the global landscape of postharvest research activity. In addition, although major citrus-exporting countries such as Spain, South Africa, Mexico, and Turkey operate within distinct regulatory, infrastructural, and logistical cold-chain environments, the peer-reviewed literature reveals a consistent global limitation: none provide longitudinal, paired temperature–quality datasets suitable for correlational analysis. Developing a detailed comparison of national cold-chain temperature monitoring systems would require primary industry reports, operational records, and grey literature that fall outside the scope of this SLR; however, we acknowledge that such region-specific assessments represent an important future research opportunity.

Secondly, keywords were structured according to the research question. Although the keywords were revised several times, studies that would have made meaningful contributions may have been excluded because they were not covered by the keywords. The studies included in this review show a geographical concentration, with a disproportionately large share originating from South Africa and European research groups. This dominance reflects both the availability of high-quality research in these regions and the strength of international collaborations centred on DT, controlled studies, and cold chain optimisation. However, it also constrains the generalisability of the findings to other production systems, climatic zones, and export environments. Thirdly, technical reports, practitioner-oriented studies, and government datasets within the postharvest logistics domain are either not indexed or available solely as grey literature, which was excluded from this review. As a result, their reference value for demonstrating the research gaps was not considered.

## 5. Conclusions and Recommendations

### 5.1. Summary of Evidence and Gap

The review synthesised 35 peer-reviewed studies on temperature-driven deterioration in citrus export supply chains and showed a methodological convergence toward controlled experiments and computational simulations (including CFD, virtual cold chains, and digital twins) with limited, sparsely instrumented commercial shipments and almost no longitudinal pairing of shipment temperature histories with arrival quality outcomes. As a result, while the literature characterises how temperature should be managed and where heterogeneities arise, it does not yet provide robust, multi-season evidence linking real shipment conditions to fruit condition on arrival. This gap constrains predictive modelling, validation of digital twins, and targeted improvements to protocol markets and logistics operations.

Few studies collected direct, standardised quality endpoints upon arrival, which prevents the assessment of temperature–quality correlations at the shipment, pallet, or position level. Together, these findings elucidate why the field has encountered challenges in transitioning from inference to evidence-based prediction under actual export conditions. The most critical gap identified is the lack of accessible, longitudinal datasets capturing both temperature and quality metrics during shipment. This hinders the development of predictive models, constrains simulation validation, and limits the optimisation of cooling strategies tailored to specific trade routes. While computational models suggest potential for energy-efficient designs, their practical applicability remains untested due to insufficient in-transit data. In addition, the underdevelopment of real-time monitoring, characterised by short temporal spans and weak quality associations, further restricts operational insights.

### 5.2. Further Work and Recommendations

This review reveals a persistent and consequential gap in citrus export logistics: the absence of longitudinal, paired shipment temperature histories and post-arrival quality outcomes. Without such datasets, predictive models cannot be robustly validated, digital twins and CFD simulations cannot be calibrated under real operating conditions, and route- or cultivar-specific operational improvements remain difficult to prioritise. Addressing this gap requires an integrated, multi-season empirical programme, improvements to sensing architecture and data governance, and regulatory alignment so that operational records are transformed into decision-quality evidence for exporters, PPECB, logistics enterprises, and receivers.

In the short term, building on the gaps identified in this review, we recommend conducting a dedicated empirical study that integrates multi-season shipment temperature profiles with arrival-quality metrics derived from commercial operations. The proposed study would analyse large-scale historical datasets spanning multiple routes, cultivars, and seasons to quantify real-world relationships between in-transit temperature deviations and postharvest outcomes. By relying on commercial shipment data rather than controlled simulations, the work would generate statistically robust evidence to validate predictive models, refine cold-chain protocols, and support evidence-based optimisation for exporters, PPECB, logistics enterprises, and receivers.

To ensure methodological rigour, arrival quality should be recorded using a harmonised assessment rubric (e.g., chilling-injury incidence and severity, decay percentage, and mass loss), with strict chain-of-custody procedures linking each quality sample to its nearest sensor stratum. This integration of temperature and quality data is essential for resolving known monitoring blind spots in the South African citrus cold chain, where evidence indicates that most temperature deviations occur during pre-cooling and loading, and where air-only sensing can fail to capture pulp temperature lag and intra-container heterogeneity. Accordingly, the pilot study should implement a hybrid sensing architecture: real-time geo-tagged cellular devices during pre-cooling, loading, and port dwell (where network coverage permits), combined with store-and-forward loggers during the sea leg, where connectivity is constrained. This layered approach strengthens data continuity across the entire logistics chain while aligning with documented findings on logger efficacy in the South African citrus export system.

In the medium term, simulation models and DT should be calibrated and validated with multi-shipment, multi-season data, moving beyond lab conditions and single-shipment case studies. Crucially, all data streams must be captured within a centralised regulatory platform so that shipment identifiers, container IDs, Schedule-1 temperature regimes, and time-stamped remote monitoring data are fully integrated for analytical validation and audit traceability. PPECB’s TITAN 2.0 already provides cold-chain value-stream modules (Export Notification, Container Inspections, Schedule 1 visibility); adding temperature-data ingestion and arrival-quality capture would convert a documentation system into a learning system. PPECB’s current compliance-oriented framework (Schedule 1 regimes, inspections, and certification) should be extended into a data-centric model by integrating continuous shipment temperature records into TITAN 2.0, standardising a minimum common data model, and systematically linking time-stamped temperature measurements to verified arrival-quality outcomes. To support this data-centric transition, a hybrid geo-tagged cellular strategy should be implemented, with real-time monitoring applied on land and store-and-forward logging used during the sea leg. This configuration utilises commercially available devices in the South African market and mitigates documented visibility gaps during pre-cooling and loading.

In the long term, we recommend institutionalising a national, multi-season repository under PPECB stewardship, with academic co-governance, to enable predictive modelling, proactive exception management, and route-level optimisation across seasons. The repository should provide controlled research access via APIs and publish annual benchmarks to enable evidence-based updates to Schedule-1 carrying regimes, pre-cooling distributions, loading/stow practices, and exception playbooks. To close the loop across the value chain, pre-harvest signals should be linked with post-harvest temperature measurements and arrival-quality outcomes to model cultivar- and region-specific susceptibility to chilling injury and decay. Collectively, these phased actions, pilot standardisation, scaled validation, regulatory integration, and national stewardship will enable predictive modelling, proactive exception management, and route-level optimisation across seasons, thereby enhancing resilience and reducing losses in South Africa’s citrus export cold chain.

## Figures and Tables

**Table 1:** Search Queries and Results Across Academic Databases.

Database	Search Query	Results
ScienceDirect	(citrus AND postharvest AND temperature) AND (“shipment records” OR “cold chain” OR “export supply chain”) AND (“quality deterioration” OR “fruit quality”)	75
Scopus	TITLE-ABS-KEY (citrus AND postharvest AND temperature) AND (“shipment records” OR “cold chain” OR “export supply chain” OR logistics OR transportation) AND (“quality deterioration” OR “fruit quality” OR “postharvest losses” OR “storage conditions”)	23
Web of Science	(citrus AND postharvest AND temperature) AND (“cold chain” OR “shipment records” OR “export supply chain”) AND (“quality deterioration” OR “fruit quality”)	12
EBSCOhost Databases (Academic Search Premier)	(Citrus AND postharvest AND temperature) AND (“cold chain” OR “shipment records” OR “export supply chain” OR logistics) AND (“quality deterioration” OR “fruit quality” OR “postharvest losses” OR “storage conditions” OR “economic impact”)	12
TOTAL		122

**Table 2:** SLR Data Extraction Sheet: Structured Framework for Citrus Quality Analysis.

Category	Subcategory	Description
Study Metadata	Author, Journal, Year	First author’s name, journal title, and publication year.
Study Scope	Geographic Focus	Country or countries where the study was conducted (e.g., South Africa or comparable citrus-exporting regions), along with primary researcher affiliations.
	Primary Research Objectives	Main goals related to postharvest deterioration of citrus quality, with an emphasis on the availability and use of historical temperature data for quality assessment.
Methodology	Analysis Type	Type of analysis employed (e.g., shipment-based using historical temperature data or experimental/simulation-based due to its absence).
	Data Sources	Sources include shipment records, empirical data, laboratory experiments, and simulations, and the study emphasises whether historical temperature data were used or whether alternative methods were required.
	Quality Assessment Techniques	Methods such as sensory evaluation, chemical analysis, or quantitative measurements, with attention to whether real-world shipment data was incorporated.
Findings	Key Results	Outcomes related to quality deterioration, temperature correlations, and data limitations, particularly in studies that lacked access to historical temperature records.
	Supply Chain Implications	Insights for management, policy, or process improvements in citrus supply chains, particularly regarding how limited historical temperature data influences conclusions.

**Table 3:** Methodological Evidence Streams Identified in the SLR.

Evidence Stream	Definition	Studies
Real-world shipment data (empirical commercial temperature records)	Studies that collect or analyse actual temperature data from commercial shipments or cold chains (e.g., via loggers like iButtons^®^, TempTale^®^, or cellular loggers), often combined with quality assessments. These provide empirical evidence of temperature breaks, variability, or non-conformances in real export conditions. Focus on frequency, location, and duration of temperature deviations (breaks/spikes) during precooling, transport to port, loading, or initial sea phase. Common issues include breaks during drenching, road transport, inspection, or container loading, risking a breach of cold sterilisation protocols. Outcomes link to potential quality defects or phytosanitary failures; however, direct fruit quality measurements upon arrival are often limited. These studies underscore real-world non-conformances; however, they rarely correlate long-term historical datasets across many shipments to detailed quality evolution.	[3,5,8,65,66,67,68,69]
Controlled experiments	Controlled experimental studies use strictly regulated laboratory or packhouse environments to isolate the specific physiological and biochemical mechanisms underlying citrus deterioration. Researchers in these studies intentionally manipulate or fix temperature regimes, such as comparing fruit performance at near-freezing −2 °C versus 10 °C, or simulating transportation at exact increments of 5 °C, 10 °C, and 15 °C, to quantify citrus responses, such as decay rates, weight loss, and rind colour changes. Many of these studies focus on providing mechanistic insights, such as evaluating how warm-temperature holding periods specifically induce flavour loss by shifting aroma volatiles, or how pre-storage heat treatments and light illumination can be used to control fungal infection and induce cold tolerance. Additionally, several studies utilise these fixed datasets to develop mathematical models for quality traits, including “temperature interruption” simulations to predict shelf-life and high-throughput phenotyping of traits like firmness and chilling injury susceptibility.	[4,14,15,43,44,70,71,72,73,74,75,76]
Computational simulations	Computational simulation-based analyses use numerical models, such as CFD, VCC, and DT, to simulate citrus cooling, heating, airflow, and quality deterioration under various postharvest and export-chain conditions. These models estimate temperature fields, heat transfer, airflow heterogeneity, cooling rates, and temperature-driven physiological responses at carton, pallet, or container scale.	[3,5,7,13,20,22,23,27,35,37,38,39,77]
Statistical and Logistics Optimisation Models	These studies use predictive statistical relationships, machine-learning models, and logistics-oriented optimisation approaches to evaluate quality outcomes, storage behaviour, or system-level decision-making.	[43,44,70,78]

**Table 4:** Comparative evaluation of emerging technologies in the citrus cold chain. Technologies are assessed according to the five SLR research questions. References in brackets correspond to studies in which the respective technology was applied, providing evidence of practical use in the literature.

Technology	QI: Temperature-Related Deterioration	QII: Use of Historical Data	QIII: Experimental vs. Simulated	QIV: Temperature–Quality Linking	Data Needs
CFD [38,39,77]	Mechanistic modelling of airflow & fruit cooling	Not required	Simulation dependent	Limited direct linkage to quality	Geometry + thermal properties; requires expertise and computing resources
VCC [7,23,29,37]	Thermal tracking via container simulations	Not required	Simulation-heavy	Limited validation against actual quality	Sensor integration, quality-linkage calibration, and validation effort
DT [3,5,13,20,22,35]	Hybrid modelling integrating physics & data	Limited by sparse historical data	Hybrid (simulation + experimental calibration)	Used for predictive quality assessment	Continuous sensor input + metadata; complex setup and integration
IoT [8,24,65,68]	Observational temperature profiling in real shipments	Core of historical datasets	Experimental, real-world deployment	Rarely directly paired with fruit quality metrics	Sensor accuracy, calibration, metadata management, maintenance and cost considerations
Machine Learning, Statistical Modelling, and Optimisation. [4,13,15,43,44,70]	Pattern extraction from temperature datasets	Requires labelled historical data	Data-driven, model training	Some correlations achievable	Large, structured datasets; computational resources; expertise in modelling

## Data Availability

Data supporting the findings of this study are available from the corresponding author upon reasonable request.

## Supplementary Materials

The following supporting information can be downloaded at: https://www.mdpi.com/article/10.3390/foods15071122/s1, PRISMA 2020 Checklist. Reference [86] is cited in the Supplementary Materials.
